# Rate of Cornea Endothelial Cell Loss and Biomechanical Properties in Fuchs' Endothelial Corneal Dystrophy

**DOI:** 10.3389/fmed.2021.757959

**Published:** 2021-11-18

**Authors:** Usanee Reinprayoon, Monthira Jermjutitham, Ngamjit Kasetsuwan

**Affiliations:** ^1^Department of Ophthalmology, Faculty of Medicine, Chulalongkorn University and King Chulalongkorn Memorial Hospital, Bangkok, Thailand; ^2^Center of Excellence for Cornea and Stem Cell Transplantation, Faculty of Medicine, Chulalongkorn University, Bangkok, Thailand

**Keywords:** FECD, corneal biomechanics, corvis, fuchs' endothelial dystrophy, corneal endothelial cell loss

## Abstract

**Background:** Our study aimed to determine the correlation between the clinical staging of Fuchs' endothelial corneal dystrophy (FECD), rate of endothelial cell loss, and corneal biomechanical properties.

**Methods:** This study combined a longitudinal retrospective/prospective analysis of corneal endothelial cell loss and a prospective cross-sectional analysis of corneal biomechanics of Fuchs' endothelial dystrophy. The trial was registered at the Thai Clinical Trials Registry as TCTR 20160927004. FECD was diagnosed by the presence of corneal guttata detected by slit lamp microscopy; the disease severity was classified into four stages using the modified Stocker's classification. *In vivo* confocal microscopy, Scheimpflug imaging, and Corneal Visualization Scheimpflug Technology were performed to evaluate endothelial cell count, central corneal thickness, and corneal biomechanical properties. Linear mixed modeling analyses were used to estimate the endothelial cell densities in a 4-year period. The corneal biomechanics were compared among the stages using Corvis ST parameters.

**Results:** Eighty eyes from eighty subjects were enrolled (42, 26, 12, and none in stages 1, 2, 3, and 4, respectively). The mean endothelial cell density was 1228.35 cells/mm^2^. The year-by-year reduction rate was 94.3 cells/mm^2^ (μ_EMM_ = −94.3, 95% CI: −115.4 to −73.2, *p* < 0.001). Corneal endothelial cell losses in Fuchs' endothelial dystrophy were estimated to be 7.7, 7.8, and 8.4% per year for stages 1, 2, and 3, respectively. The mean corneal thicknesses of stages 1, 2, and 3 were 556 ± 32, 623 ± 33, and 648 ± 50 mm, respectively. For the corneal biomechanical parameters, the A1-length and A1-time were significantly different between stages 1 and 3 (A1-length: mean diff_stage1vs.3_ = 0.10, 95% CI: < 0.001–0.15, *p* < 0.001, A1-time: mean diff_stage1vs.3_ = −0.24, 95% CI: −0.41 to −0.07, respectively).

**Conclusions:** In the advanced stage, corneas significantly changed their biomechanical viscoelastic behavior by decreasing resistance, as measured by a longer A1-length and shorter A1-time.

## Introduction

Fuchs' endothelial corneal dystrophy (FECD) is characterized by thickened Descemet's membrane, guttae formation, and progressive loss of corneal endothelial cells. This causes corneal swelling, and the cornea becomes opaque in the advanced stages of the disease. The standard treatment for significant visual disturbance in FECD is endothelial transplantation (e.g., Descemet stripping automated endothelial keratoplasty or Descemet membrane endothelial keratoplasty). The central corneal endothelial cell density (ECD) declines at a rate of 0.6% per year in normal physiology (1). Cross-sectional studies have shown variable rates (0.8–3% per year) of corneal endothelial cell loss in patients with FECD (2, 3).

Corneal biomechanics include corneal elasticity and stiffness. Most researchers focus on this property in ectatic corneal diseases, which have abnormal collagen and extracellular matrix. Their corneas are usually thinner and have weaker biomechanical properties than those with normal corneal tissue (4). The pathophysiology of FECD leads to a decrease in the number of endothelial cells, causing corneal swelling that affects vision and may result in corneal endothelial decompensation. Changes in corneal tissue hydration might interfere with the stiffness property. Therefore, corneal biomechanical changes might be an earlier sign of FECD rather than a clinical presentation of corneal edema. To date, there have been limited studies focusing on the corneal biomechanics in FECD (5, 6).

This study aimed to determine the rate of endothelial cell loss using longitudinal data, which provides information on disease progression, and investigate corneal biomechanical properties at different stages of severity in patients with FECD.

## Materials and Methods

This study was a longitudinal retrospective/prospective analysis of ECD and a prospective cross-sectional analysis of corneal biomechanical properties in FECD. This study followed the tenets of the Declaration of Helsinki and was approved by the Institutional Review Board of the Faculty of Medicine, Chulalongkorn University (COA No. 594/2016) and registered at the Thai Clinical Trials Registry (TCTR 20160927004). Informed consent was obtained from all participants.

The corneal dystrophy database at King Chulalongkorn Memorial Hospital was searched to identify patients who had FECD diagnosed by cornea specialists between 2008 and 2017, and their medical records were reviewed. All reviewed patients were invited to participate in the study by phone. Only the patients who agreed to participate and provided informed consent were enrolled in the study. Patients with a history of intraocular trauma, laser peripheral iridotomy, corneal infection, previous corneal transplantation, contact lens wearers, or patients who could not undergo investigations were excluded from the study. In cases in which both eyes of a patient qualified for inclusion, only the right eye was included. The collected data included age, sex, family history of FECD, underlying systemic diseases, presence of corneal edema, presence of corneal endothelial pigment deposits, history of previous cataract surgery, ECD, and central corneal thickness (CCT). On the visit date, non-contact computerized tonometer and slit-lamp examinations were performed to determine the extent of corneal guttae, presence of corneal edema, and subepithelial scarring to grade the disease severity according to an established 4-scale severity grading system (modified Stocker's classification) (7):

stage 1—corneal guttae without epithelial or stromal edema.
stage 2—corneal guttae with epithelial or stromal edema.
stage 3—corneal edema associated with scar formation.
stage 4—corneal opacification with neovascularization.

Three non-invasive instruments were employed by an experienced technician: *in vivo* confocal microscopy (Confoscan 4, Nidek Technologies, Albignasego, Italy), Corneal Visualization Scheimpflug Technology (Corvis ST, Oculus Optikgeräte, Inc., Wetzlar, Germany), and Scheimpflug imaging (Pentacam, Oculus, Wetzlar, Germany) were used to measure ECD, biomechanical properties, and CCT, respectively.

### *In vivo* Confocal Microscopy (Confoscan 4)

A Confoscan 4 microscope with a 40x objective lens was used to image corneal endothelial cells; the best quality images with a region of interest > 0.05 mm^2^ were chosen. Automated analysis by the manufacturer's software (NAVIS) was traced. If the automated software failed to correctly identify the cell borders, the blinded technician will perform manual cell identification at the center of each cell. Subsequently, the manufacturer's software calculated and reported ECD. If no endothelial cells were identified, the sample was defined as uncountable.

### Corneal Visualization Scheimpflug Technology (Corvis ST)

Scheimpflug technology was used to automatically record corneal dynamics at 4,300 frames per second after deformation by a collimated air puff in the sitting position. To assess the corneal biomechanics, the Scheimpflug images were classified into three states: applanation 1 (A1) or the flattened state, while the cornea rebounded inward; highest concavity (HC); and applanation 2 (A2) or the flattened state, while the cornea rebounded outward. In each state, Corvis ST measured the length of the flattened cornea (A1-length and A2-length), elapsed time (A1-time, HC time, and A2-time), and velocity (A1-velocity and A2-velocity). In the HC state, the deformation amplitude (DA, the distance of the corneal apex from the initial state to the highest concavity), peak distance (the distance between two apexes at HC), and HC radius (the radius of curvature of the cornea at the time of maximum concavity) were calculated. These 10 Corvis ST parameters are critical for the evaluation of corneal biomechanical properties.

### Scheimpflug Imaging (Pentacam)

Pentacam scans automatically when patients directly fixate on a target light. Within a couple of minutes, the CCT was obtained using apical corneal pachymetry.

### Statistical Analysis

All patient characteristics, including age, sex, underlying systemic diseases, family history of FECD, time of EC counts, extent of guttae area, presence of corneal endothelial pigment deposits, history of previous cataract surgery, intraocular pressure, and CCT were analyzed using means and standard deviations for continuous variables and counts and percentages for categorical variables. The data were analyzed for normal distribution using the Shapiro–Wilk test.

Linear mixed modeling analysis was used to estimate the corneal ECD over time and between each stage of FECD. This model was chosen because the ECD of each patient's eye was repeatedly measured over time. Both bivariate and multivariate analyses were performed to estimate unadjusted and adjusted effects. All predictors with *p* < 0.25 in the bivariate models were considered sufficiently important for consideration in the multivariable model. The exception to this rule was ECD, the study effect, which was forced into the multivariable model regardless.

To analyze the corneal biomechanical parameters, general linear modeling was used to fit both the continuous and categorical predictors of each corneal biomechanical parameter throughout all analyses. Statistical significance was set at *p* < 0.05. All analyses were performed using the R program version 3.4.1 (2017) (The R Foundation, Boston, MA, USA).

## Results

There were 164 patients enrolled; however, only 80 patients met the inclusion criteria. There were 80 eyes from 80 patients; 63 (79%) were female and 17 (21%) were male, with a mean age of 61.9 ± 11.1 years (range: 21–84 years). In the 4-stage clinical severity grading system, 42 (52%), 26 (33%), and 12 (15%) patients were in stages 1, 2, and 3, respectively. No patients were categorized as stage 4. The demographic data, corneal characteristics, and CCT are summarized in Table 1. The median follow-up time was 4 years (range: 0–9.2 years). Each patient was examined annually; however, *in vivo* confocal microscopy was not performed at every visit. The mean ECD at baseline was 1228.35 cells/mm^2^, and 11 subjects had uncountable cells at the first presentation.

**Table 1:** Demographic data and corneal characteristics.

**Characteristic**	**Total**	**Stage 1**	**Stage 2**	**Stage 3**
Number, *n*	80	42	26	12
Age, y ± SD	61.9 ± 11.1	62 ± 10	61.6 ±12.5	62.6 ± 12.7
Female, *n*	63	34	21	8
Diabetes, *n*	13	7	4	2
Hypertension, *n*	30	18	9	3
Dyslipidemia, *n*	18	12	5	1
Family history, *n*	24	10	12	2
**Times of cell count**, ***n***				
1	24	14	8	2
2	29	13	11	5
3	17	8	5	4
4	6	5	1	0
5	4	2	1	1
**Guttata location**, ***n***				
Center	51	30	17	4
Diffuse	26	12	9	8
**Pigment deposits**, ***n***				
Presence	45	19	20	6
Absence	35	23	6	6
**Lens type**, ***n***				
Crystalline lens	54	8	20	8
Pseudophakia	26	16	6	4
IOP, mmHg ± SD	–	11 ± 3	11 ± 3	9 ± 3
CCT, μm ± SD	–	556 ± 32	623 ± 33	648 ± 50

*Data shows the mean ± SD. IOP, intraocular pressure; CCT, central corneal thickness*.

### Primary Outcome

The linear mixed model revealed that we would expect ECD to decrease by 94.32 cell/mm^2^ (μ_EMM_ = −94.32, 95% CI: −115.44 to −73.20, *p* < 0.001) for each year following diagnosis. Although there were some differences in the reduction in ECD with stage levels (μ_EMM_ = −9.34, 95% CI: −188.11 to 169.44, *p* = 0.92 in stage 2 and μ_EMM_ = −88.18, 95% CI: −405.86 to 229.49, *p* = 0.59 in stage 3), the differences were not statistically significant. Of the factors (family history, underlying hypertension or dyslipidemia, and previous cataract surgery) that were included in the multivariable model, the presence of family history tended to have an effect on ECD: on average, 237.39 cell/mm^2^ per year fewer than those without a family history (95% CI: −606 to 131.37, *p* = 0.21); however, this was not statistically significant (Table 2).

**Table 2:** Corneal endothelial cell reduction per year and comparison between stages.

**Effect**	**Unadjusted**		**Adjusted**		
	**X^2^LRT = 0.27**, ***p*** **= 0.88**		**X^2^LRT = 0.92**, ***p*** **= 0.63**		
	**Estimate (cell/mm^2^)**	***P*** **-value**	**Estimate (cell/mm^2^)**	***P*** **-value**	**95% CI**
**Stage**					
1 (Intercept)	1251.93	–	1216.82	–	962.87–1470.77
2	−13.02	0.89	−9.34	0.92	−188.11 to 169.44
3	−84.54	0.6	−88.18	0.59	−405.86 to 229.49
Yearly	−89.75	<0.001	−94.32	<0.001	−115.44 to −73.20
Family history	−324.29	0.08	−237.39	0.21	−606.14 to 131.37
Female	−209.97	0.32	–	–	–
Diffuse guttata	−122.22	0.4	–	–	–
Increasing age	8.68	0.25	–	–	–
Presence of pigment deposits	25.84	0.81	–	–	–
Previous cataract surgery	101.30	0.24	76.12	0.38	−94.23 to 246.48
Diabetes	115.14	0.59	–	–	–
Hypertension	229.76	0.17	51.77	0.79	−328.28 to 431.82
Dyslipidemia	352.45	0.06	263.61	0.23	−164.01 to 691.22

The estimated marginal means (or least squares mean) for each stage are shown in Table 3.

**Table 3:** Estimated marginal mean of each stage.

**Stage**	**lsmean (cell/mm^2^)**	**Standard error**	**95% CI**
1	1105.31	111.23	883.52– 1327.10
2	1095.97	127.38	843.26–1348.68
3	1017.12	184.8	651.32–1382.93

*lsmean, least square mean or estimated marginal mean*.

### Secondary Outcome

The 10 corneal biomechanical parameters recorded by Corvis ST are outlined in Table 4. The general linear model suggested that the A1-length in both stages 2 and 3 differed from that of stage 1. When we accounted for potential confounders (CCT), at least two stages were different (*F* = 18.24, df = 3, df = 76, *p* < 0.001). The *post hoc* comparisons revealed that only stage 3 had a significantly longer A1-length than stage 1 (mean difference_stage1vs.3_ = 0.10, 95% CI: < 0.001–0.15, *p* < 0.001).

**Table 4:** Corneal biomechanical properties measured by Corvis ST.

**Parameters**	**Stage 1 (*n* = 42)**	**Stage 2 (*n* = 26)**	**Stage 3 (*n* = 12)**	**Unadjusted**		**Adjusted**	
				**Diff.^a^ (p)**	**Diff.^b^ (p)**	**Diff.^c^ (p)**	**Diff.^d^ (p)**
A1-Length, mm	1.79 ± 0.05	1.84 ± 0.05	1.92 ± 0.09	0.06 (<0.001)	0.13 (<0.001)	0.04 (0.07)	0.10 (<0.001)
A1-Time, ms	6.89 ± 0.25	6.83 ± 0.23	6.66 ± 0.39	−0.07 (0.31)	−0.24 (<0.01)	−0.08 (0.24)	−0.24 (<0.01)
A1-Velocity, m/s	0.14 ± 0.01	0.14 ± 0.02	0.16 ± 0.03	0.00 (0.79)	0.01 (0.07)	0.00 (0.88)	0.01 (0.10)
HC-Time, ms	17.28 ± 0.47	17.39 ± 0.49	17.29 ± 0.31	0.10 (0.37)	0.00 (0.99)	0.10 (0.38)	0.00 (0.99)
Peak distance, mm	2.37 ± 0.15	2.37 ± 0.15	2.47 ± 0.12	−0.01 (0.88)	0.09 (0.06)	−0.00 (0.99)	0.09 (0.05)
HC Radius, mm	6.45 ± 0.62	6.46 ± 1.32	6.49 ± 0.76	0.01 (0.96)	0.04 (0.90)	−0.01 (0.95)	0.01 (0.98)
DA, mm	1.18 ± 0.12	1.19 ± 0.1	1.2 ± 0.15	0.01 (0.79)	0.02 (0.68)	0.05 (0.23)	0.07 (0.22)
A2-Length, mm	1.64 ± 0.35	1.86 ± 0.31	2.02 ± 0.28	0.21 (0.01)	0.37 (<0.001)	0.06 (0.59)	0.19 (0.19)
A2-Time, ms	21.87 ± 0.37	21.89 ± 0.33	21.89 ± 0.54	0.01 (0.90)	0.01 (0.93)	−0.02 (0.87)	0.05 (0.68)
A2-Velocity, m/s	−0.41 ± 0.07	−0.38 ± 0.07	−0.38 ± 0.06	0.03 (0.13)	0.03 (0.26)	0.01 (0.74)	0.00 (0.93)

*Data shows the mean ± SD. General linear analysis was performed to test differences (Diff.) between three groups. Bivariate analysis:*

a *stage 1 vs. stage 2;*

b *stage 1 vs. stage 3.*

c *Multivariate analysis: stage 1 vs. stage 2;*

d *stage 1 vs. stage 3.*

*A1-length, cord length of applanation 1; A1-time, time from starting until applanation 1; A1-velocity, the speed of applanation 1; HC-time, time from starting until highest concavity; Peak distance, distance of the apexes at highest concavity; HC Radius, central concave curvature at highest concavity; DA, maximum deformity amplitude at the corneal apex; A2-length, cord length of applanation 2; A2-time, time from starting until applanation 2; A2-velocity, the speed of applanation 2*.

There was a statistically significant difference in A1-time between stages 1 and 3. When the adjusted models (age and lens) were applied, at least two stages differed (*F* = 3.50, df = 4, df = 75, *p* = 0.01). The analysis of the individual differences showed that stage 3 differed from stage 1 (mean diff = −0.24, 95% CI: −0.41 to −0.07).

The A2-length in clinical stages 1, 2, and 3 increased with statistical significance (*F* = 7.35, df = 2, df = 77, *p* < 0.01). Stage 2 had an average of 0.21 mm longer A2-length than stage 1 (95% CI: 0.05–0.38, *p* = 0.01). Stage 3 had an ~0.37 mm longer A2-length than stage 1 (95% CI: 0.16–0.59, *p* < 0.001). After accounting for potential confounders (sex, family history, previous cataract surgery, and CCT), no statistically significant difference was noted (stage 2: *p* = 0.59, stage 3: *p* = 0.19).

## Discussion

After adjusting for important confounders (including age, family history of FECD, and previous cataract surgery) for each year since diagnosis, the rates of ECD loss in our study (7.7, 7.8, and 8.4% per year in FECD stages 1, 2, and 3, respectively) were greater than those in a previous study by Hatou et al. (2) in which ECD losses were 0.81, 2.65, and 3.08% in stages 1, 2, and 3, respectively. This may be due to differences in population, the ECD measurement technique, the statistical analysis method, and the number of subjects. Their study used cross-sectional data, which assumes that all variations are between subjects; however, ECD decreases occur within individual subjects over time. We used linear mixed modeling to determine ECD loss, which is a model for longitudinal outcomes that can be adjusted with other covariates to reduce error and minimize bias. A previous study formulated a mathematical model for predicting the decrease in endothelial cell number by assuming that the ECD of the cornea is 3,600 cells/mm^2^ at the age of 5 years and decreases at a constant rate. However, for the onset of FECD in adults, these assumptions may not be correct. Furthermore, a statistical difference in ECD loss by stage was not observed in our study.

Fifteen subjects had uncountable ECD on some visits and were, therefore, not included in the analysis. Of these, 6 patients were in stage 1, indicating that even though the cell structure at the center of the cornea (0.05 mm^2^ zone) was altered, the remaining endothelial cells were still able to adequately function. This suggests that corneal thickness may be a better indirect indicator of corneal endothelial cell function. Following this concept, Sun et al. (8) used Scheimpflug tomography to determine subclinical edema in FECD and classify the disease severity.

Corneal biomechanical properties are novel parameters that were found to change depending on disease severity. The reduction of the corneal endothelial cell number results in decreasing pump function (9). Greater water content in the corneal tissue could disrupt the complicated architecture of the stroma, which can cause the cornea to become flaccid.

In our study, all corneal biomechanical parameters, except HC-time, correlated with the clinical staging. We found that FECD in clinical stage 3 had a shorter A1-time, longer A1-length, longer A2-length, longer HC radius, and greater deformation amplitude than FECD in stage 1. Only the A1-length and A1-time were statistically significant between stages 1 and 3. These results indicate that corneas in FECD stage 3 took a shorter time to be applanated, suggesting that corneas with more compromised endothelial function are more easily compressed by collimated air during the inward and outward periods. In other words, the cornea becomes less resistant as the severity of FECD becomes more advanced. These results are different from those of keratoconus eyes, whose abnormal corneal viscoelasticity is characterized by a shorter A1-time, shorter HC radius, longer A2-time (rebound time), increased A2-velocity, and greater deformity amplitude (4). The differences in the A1-length and A1-time between the stages of FECD suggest that the A1-length and A1-time are potential parameters for monitoring disease progression and for prognostic prediction.

This study had some limitations. First, some necessary information may not be recorded owing to the retrospective data for endothelial cell loss analysis. Second, there was no control group to avoid any bias.

In conclusion, this was the first longitudinal study to demonstrate ECD loss at each stage of FECD severity. Furthermore, it measured corneal biomechanical transformation in FECD by comparing corneal viscoelastic properties at each stage of corneal severity. Our findings provide a better understanding of the natural history, pathophysiology, and prognosis of FECD and can inform more specific and appropriate treatment plans.

## Data Availability Statement

The raw data supporting the conclusions of this article will be made available by the authors, without undue reservation.

## Ethics Statement

The studies involving human participants were reviewed and approved by the Institutional Review Board of Faculty of Medicine, Chulalongkorn University (COA No. 594/2016). The patients/participants provided their written informed consent to participate in this study.

## Author Contributions

UR, MJ, and NK devised the idea of the methodology, drafted the protocol, and proofread the manuscript. MJ conducted the literature review and drafted the manuscript. UR and NK provided verification and editing. All authors contributed to the article and approved the submitted version.

## Funding

This work was supported by the Ratchadapiseksompotch Fund, Faculty of Medicine, Chulalongkorn University (grant number RA 59/084).

## Conflict of Interest

The authors declare that the research was conducted in the absence of any commercial or financial relationships that could be construed as a potential conflict of interest.

## Publisher's Note

All claims expressed in this article are solely those of the authors and do not necessarily represent those of their affiliated organizations, or those of the publisher, the editors and the reviewers. Any product that may be evaluated in this article, or claim that may be made by its manufacturer, is not guaranteed or endorsed by the publisher.
